# Interleukin-21 signaling in B cells, but not in T cells, is indispensable for the development of collagen-induced arthritis in mice

**DOI:** 10.1186/s13075-016-1086-y

**Published:** 2016-08-17

**Authors:** Koji Sakuraba, Akiko Oyamada, Kenjiro Fujimura, Rosanne Spolski, Yukihide Iwamoto, Warren J. Leonard, Yasunobu Yoshikai, Hisakata Yamada

**Affiliations:** 1 Division of Host Defense, Medical Institute of Bioregulation, Kyushu University, 3-1-1 Maidashi Higashi-ku, Fukuoka, 812-8582 Japan; 2 Clinical Research Institute, National Hospital Organization, Kyushu Medical Center, Fukuoka, Japan; 3 Department of Orthopaedic Surgery, Kyushu University, Fukuoka, Japan; 4 Laboratory of Molecular Immunology, National, Heart, Lung, and Blood Institute (NHLBI), Bethesda, MD USA

**Keywords:** Interleukin-21, Collagen-induced arthritis, Rheumatoid arthritis, Cytokine, B cell

## Abstract

**Background:**

Interleukin-21 (IL-21) is a T-cell-derived cytokine whose receptor is expressed on a variety of cells and therefore might have pleiotropic roles in the pathogenesis of rheumatoid arthritis (RA). In this study, we investigated the involvement of IL-21 signaling in the development of collagen-induced arthritis (CIA), an animal model of RA, using IL-21 receptor knockout (*Il21r* KO) mice.

**Methods:**

*Il21r* KO mice or wild-type (WT) C57BL/6 mice were immunized with chicken type II collagen (CII) emulsified in complete Freund adjuvant on day 0 and were given a boost injection on day 21. The production of anti-CII antibody, development of T-cell and B-cell subsets, and T-cell responses to CII were analyzed. CIA was induced in *Rag2* KO mice to which combinations of WT or *Il21r* KO CD4 T cells and WT or *Il21r* KO B cells had been transferred, in order to examine the role of IL-21 signaling in each cell subset.

**Results:**

*Il21r* KO mice were resistant to the development of CIA. CII-specific IgG but not IgM production was impaired in *Il21r* KO mice. This is consistent with a reduction of germinal center B cells in the draining lymph nodes. In contrast, CII-specific Th1 and Th17 responses were unaffected in *Il21r* KO mice. There was also no difference in the number of CII-specific follicular helper T cells between WT and *Il21r* KO mice. By analyzing the development of CIA in T-cell and B-cell mixed transfer experiments, we confirmed that IL-21 receptor expression on B cells, but not on T cells, was essential for the development of CIA.

**Conclusion:**

IL-21 signaling in B cells, but not in T cells, plays essential roles in the production of pathogenic autoantibodies that induce CIA development.

## Background

Interleukin-21 (IL-21) is a member of the common cytokine receptor γ-chain family of cytokines and is produced primarily by activated T cells, especially by follicular helper T (Tfh) cells, natural killer T (NKT) cells, and T-helper cell type 17 (Th17) cells [1]. The IL-21 receptor (IL-21R) is expressed on a variety of cells, including nonhematopoietic cells, and therefore IL-21 has a wide range of biological functions [1]. In CD4 T cells, IL-21 promotes the development of Th17 and Tfh cells [2–4], while in B cells it is involved in maintenance, class switching, and somatic hypermutation [5–7].

Rheumatoid arthritis (RA) is characterized by chronic inflammation of multiple joints, which is accompanied by the production of autoantibodies, such as rheumatoid factor (RF) and anti-citrullinated protein antibody (ACPA). IL-21R was upregulated in RA synovium [8] while an increased frequency of IL-21-producing T cells and high concentrations of IL-21 were observed in the synovial fluid or serum of RA patients [9–12]. A genetic association between IL-21 and RA has also been reported [13]. IL-21 might therefore be involved in the pathogenesis of RA, possibly via the induction of pathogenic T-cell responses and/or autoantibody production.

Collagen-induced arthritis (CIA) is a widely used animal model of RA, and CIA can be induced by immunization of mice (or rats) with type II collagen (CII). Many pathological aspects of CIA resemble human RA. Most, if not all, RA patients exhibit T-cell and B-cell responses to CII [14, 15]. Similar to human RA, the MHC class II haplotype strongly influences the susceptibility to CIA in mice, indicating the importance of CD4 T-cell responses in pathogenesis [16, 17]. In fact, CD4 T-cell depletion before immunization prevented the development of CIA [18, 19]. The development of CIA also depends on anti-CII antibody (Ab) production because B-cell-deficient mice are totally resistant to the induction of CIA [19, 20]. Thus, one of the roles of CD4 T cells in the development of CIA is to help Ab production by B cells. In addition, IL-17-producing CD4 T cells (i.e., Th17 cells) are also implicated in the development of CIA [21, 22].

Since IL-21 regulates the development of Th17 cells, Tfh cells, as well as Ab-producing B cells, IL-21 is implicated in the development of CIA. In fact, blocking IL-21 by administration of IL-21R-Fc after the onset of CIA ameliorated the ongoing disease [23, 24]. However, the detailed roles of IL-21 in the immune responses involved in the development of CIA are yet to be determined. In this study, we induced CIA in IL-21 receptor knockout (*Il21r* KO) mice to analyze the roles of IL-21 signaling in the induction of arthritogenic T-cell and B-cell responses in CIA.

## Methods

### Mice

Wild-type (WT) C57BL/6 mice were purchased from Charles River Japan (Yokohama, Japan). The generation of *Il21r* KO mice was described previously [7]. *Rag2* KO mice were purchased from CREA Japan (Tokyo, Japan). The mice were bred under specific pathogen-free conditions in our institute and were used for the experiments at 6–12 weeks of age.

### Induction and assessment of CIA

Mice were immunized s.c. with 200 μg of chicken CII (Collagen Research Center, Tokyo, Japan) emulsified in 50 μl Freund’s complete adjuvant (CFA) containing 250 μg of *Mycobacterium tuberculosis* H37RA (DIFCO, Detroit, MI, USA). Mice were boosted 3 weeks later with 200 μg of CII emulsified in 50 μl CFA. The development of arthritis was evaluated three times a week, and the severity of arthritis was scored as follows: 1 point was assigned to an inflamed (showing redness and/or swelling) digit, mid paw, or ankle/wrist, but 2 points were assigned to digits if more than one digit was inflamed. The sum of these points was the score of each paw, and therefore the maximum score was 4. The total score per mouse ranged from 0 to 16.

### Histological evaluation by hematoxylin and eosin staining

Mouse hind limbs were removed and the skin peeled off before fixation with 10 % neutral buffered formalin. After decalcification with 5 % formic acid, the samples were embedded in paraffin and cut into 3 μm thick sections, which were mounted on glass slides and stained with hematoxylin and eosin.

### Measurement of serum anti-CII Ab levels

Serum levels of anti-CII Abs were measured by enzyme-linked immunosorbent assay (ELISA). Briefly, microtiter plates were coated with chicken CII (10 μg/ml) overnight at 4 °C. After washing and blocking, serum samples were added in serial dilutions and incubated for 2 h at room temperature. After four washes, peroxidase-conjugated goat anti-mouse IgG (KPL, Baltimore, MD, USA), rabbit anti-mouse IgG1 (Invitrogen, Carlsbad, CA, USA), IgG2c (Invitrogen), or biotin-conjugated anti-mouse IgM (II/41; eBioscience, San Diego, CA, USA) was added and incubated for 2 h at room temperature. For the anti-mouse IgM, streptavidine–HRP (R&D System, Minneapolis, MN, USA) was added after four washes and incubated for 30 min at room temperature. Ab binding was visualized using TMBS (eBioscience).

### Antibodies and flow cytometric analysis

FITC-conjugated anti-GL7 (GL7) and anti-CD278 (ICOS; C398.4A) mAbs were purchased from BioLegend (San Diego, CA, USA). Alexa Flour 488-conjugated anti-IL-17A (TC11-18H10) mAb, allophycocyanin-conjugated anti-CD45R (RA3-6B2) and anti-CD4 (RM4-5) mAbs, PE-conjugated CD95 (Jo2) mAbs and streptavidin, PerCP-Cy5.5-conjugated anti-CD19 (1D3) and anti-IFNγ (XMG1.2) mAbs, and biotin-conjugated anti-CD185 (CXCR5; 2G8) mAbs were purchased from BD Biosciences (San Jose, CA, USA). PE-conjugated anti-CD154 (MR1) mAbs and PerCP-Cy5.5-conjugated anti-IFNγ (XMG1.2) mAbs were purchased from eBioscience. For cell surface staining, a single-cell suspension was incubated with the optimal concentration of fluorescent mAbs for 20 min at 4 °C. Intracellular staining was performed using the BD Cytofix/Cytoperm Kit (BD Biosciences) according to the manufacturer’s instructions. Stained cells were run on a FACSCalibur flow cytometer (BD Biosciences). In some experiments, we added propidium iodide (1 μg/ml) to the cell suspension just before running on the flow cytometer to detect and exclude dead cells for the analysis. The data were analyzed using BD CellQuest software Version 3.3 (BD Biosciences).

To detect antigen-specific T cells, intracellular CD154 expression was examined after ex-vivo stimulation with the antigens as described previously [25]. Briefly, the draining (inguinal) lymph node (LN) cells were cultured for 18 h at 37 °C with denatured CII (100 μg/ml) or purified protein derivative (PPD, 10 μg/ml; Japan BCG Laboratory, Tokyo, Japan). Brefeldin A (10 μg/ml; Sigma-Aldrich, St. Louis, MO, USA) was added to the culture medium for the last 4 h. The cell culture medium used in this study was RPMI 1640 (Wako Pure Chemical Industries, Osaka, Japan) supplemented with 10 % FBS (Cell Culture Technologies, Gravesano, Switzerland), 100 U/ml penicillin, 100 mg/ml streptomycin, and 0.5 mM 2-mercaptoethanol.

### Measuring cytokine production of LN cells

Five weeks after immunization, the draining LN cells were harvested and stimulated with denatured CII (100 μg/ml) or PPD (10 μg/ml) at 1 × 10^7^/well in 96-well flat-bottomed plates for 72 h. Interferon gamma (IFN-γ) and IL-17 production in the culture supernatant was measured using ELISA kits (DuoSet; R&D System) according to the manufacturer’s instructions.

### Adoptive transfer of T cells and B cells into RAG2 KO mice

CD4 T cells were purified from the draining LN of WT mice or *Il21r* KO mice 14 days after immunization with CII/CFA using anti-CD4 microbeads and an autoMACS cell separator (Miltenyi Biotec, Bergisch Gladbach, Germany) according to the manufacturer’s instructions. B cells were purified from the LNs and the spleens of naïve WT mice or *Il21r* KO mice using PE-conjugated anti-CD19 mAb (1D3; BD Bioscience) and anti-PE microbeads (Miltenyi Biotec). The purity of both CD4^+^ T cells and CD19^+^ B cells was greater than 95 %. The purified CD4^+^ T cells (3 × 10^7^/μl) and B cells (1 × 10^8^/μl) were injected i.v. into *Rag2* KO mice, which were subsequently immunized with chicken CII and CFA to induce CIA. We confirmed the presence of the transferred CD4^+^ T cells and CD19^+^ B cells in the peripheral blood of recipient RAG2 KO mice by flow cytometry prior to CII immunization.

### Statistics

Statistical significance was calculated using the log-rank test, one-way ANOVA with a Turkey’s multiple comparison test, and two-way repeated-measure ANOVA by Prism software version 4.0a (GraphPad Software). *p* < 0.05 was considered statistically significant.

## Results

### *Il21r* KO mice are resistant to the induction of CIA

**Fig. 1:**
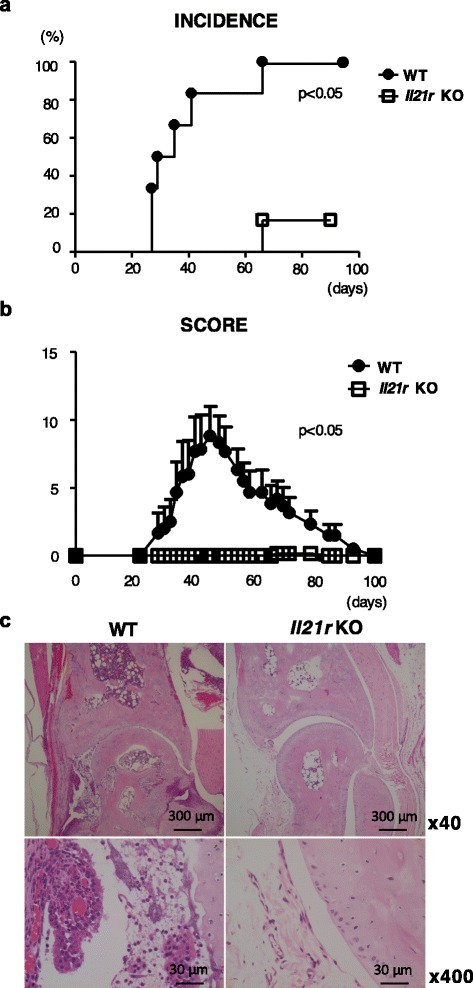
*Il21r* KO mice were resistant to CIA. Mice were immunized with chicken CII emulsified in CFA and were boosted 3 weeks later. Incidence (**a**) and severity score (**b**) of CIA in WT mice (*filled circle*, *n* = 6) or *Il21r* KO mice (*open square*, *n* = 6). Clinical scores were calculated only in affected mice. Each point represents mean ± SEM. Statistical significance was analyzed by the log-rank test (**a**) or two-way ANOVA with repeated measures (**b**). *p* < 0.05, different between WT mice and *Il21r* KO mice. Data are representative of three independent experiments. **c** Histological examination of the hind limb from WT mice (*left panel*) or *Il21r* KO mice (*right panel*) 50 days after immunization. The samples were stained with hematoxylin and eosin (magnification: *upper*, ×40, *lower*, ×400). Il21r *KO* IL-21 receptor knockout, *WT* wild type

*Il21r* KO mice or WT mice were immunized with CII emulsified with CFA, followed by a booster injection after 3 weeks. Arthritis started to develop in WT mice about 1 week after the boost whereas *Il21r* KO mice were highly resistant to the development of arthritis (Fig. 1a, b). Histological examination showed no cellular infiltrates in the joints of *Il21r* KO mice, while massive synovitis with cartilage and bone destruction was detected in WT mice (Fig. 1c).

### Impaired CII-specific Ab production in *Il21r* KO mice

**Fig. 2:**
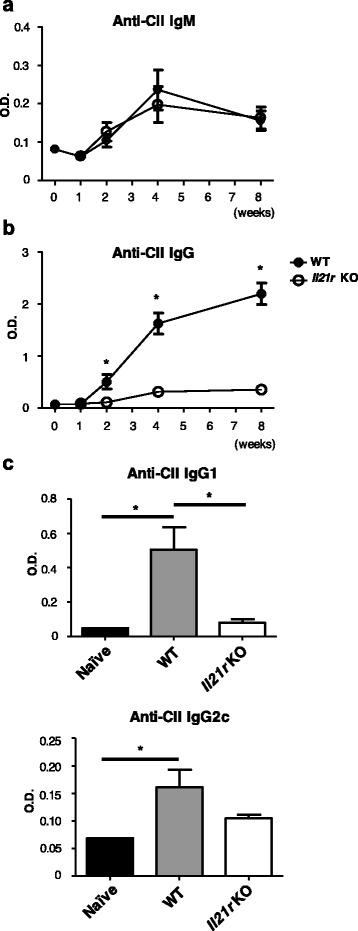
CII-specific IgG production was suppressed in *Il21r* KO mice with CIA. Levels of anti-CII IgM (**a**) and IgG (**b**) Abs in the serum of WT mice (*filled circle*, *n* = 6) or *Il21r* KO (*open circle*, *n* = 6) mice during the course of CIA. **c** Levels of anti-CII IgG1 (*upper panel*) and IgG2c (*lower panel*) Abs in the serum of WT mice (*gray column*, *n* = 6) and *Il21r* KO mice (*open column*, *n* = 6) at 5 weeks after CIA induction. Sera from naïve WT mice were used as the negative control (*filled column*, *n* = 6). Levels of anti-CII IgG1 and IgG2c Abs in the serum of naïve *Il21r* KO mice were not different from those of naïve WT mice. Error bars represent mean ± SEM. Statistical significance was analyzed by two-way ANOVA with repeated measures (**a**, **b**) or one-way ANOVA with Tukey’s multiple comparison test (**c**). *p < 0.05, different between WT mice and *Il21r* KO mice or naïve and immunized mice. Data are representative of three independent experiments. *CII* type II collagen, Il21r *KO* IL-21 receptor knockout, *O.D.* optical density, *WT* wild type

To elucidate the mechanism of CIA resistance in *Il21r* KO mice, we measured anti-CII Abs in the serum. Although there was no significant difference in the levels of anti-CII IgM between WT mice and *Il21r* KO mice, CII-specific IgG production, which was detected from 2 weeks after immunization, was severely impaired in *Il21r* KO mice (Fig. 2a, b). There was a significant decrease of CII-specific IgG1, while CII-specific IgG2c tended to decrease in *Il-21r*KO mice (Fig. 2c). CII-specific IgG2c was not different between naïve and immunized *Il21r* KO mice (Fig. 2c).

**Fig. 3:**
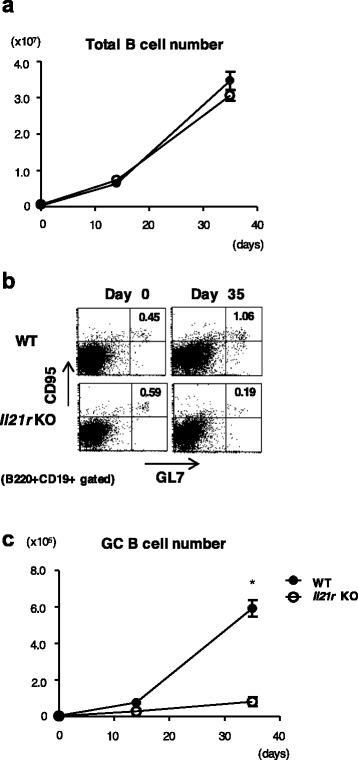
Germinal center (*GC*) B-cell responses were suppressed in *Il21r* KO mice with CIA. **a** Absolute numbers of total B cells (CD19^+^CD45R^+^ cells) of the draining LNs in WT mice (*filled circle*) or *Il21r* KO mice (*open square*) during the course of CIA. **b** Representative dot plots of GL7 and CD95 expression on B cells gated by B220 and CD19 at 0 or 35 days after CIA induction. Numbers (*upper right quadrant*) indicate the percentage of CD95^+^GL7^+^ cells in B cells (CD19^+^B220^+^ cells). **c** Absolute numbers of GC B cells (GL7^+^CD95^+^CD19^+^B220^+^ cells) in the draining LNs of WT mice (*filled circle*) or *Il21r* KO mice (*open circle*) during the course of CIA. Error bars represent mean ± SEM. Statistical significance was analyzed by two-way ANOVA with repeated measures (**a**, **c**). *p < 0.05, different between WT mice and *Il21r* KO mice. Data are representative of three independent experiments. Il21r *KO* IL-21 receptor knockout, *WT* wild type

IL-21 has been shown to be involved in germinal center (GC) reactions [5]. Thus, we compared the number of GC B cells in the draining LNs of WT mice and *Il21r* KO mice. There was no difference in the number of total B cells between WT mice and *Il21r* KO mice at any time points (Fig. 3a). Although the numbers of GC B cells, which were identified by the expression of GL7 and CD95, were also comparable between WT mice and *Il21r* KO mice before immunization, GC B cells increased after immunization with CII only in WT mice (Fig. 3b, c). IL-21 signaling is thus important for the generation and/or expansion of GC B cells after antigen immunization.

### Normal clonal expansion and cytokine production of antigen-specific T cells in *Il21r* KO mice

**Fig. 4:**
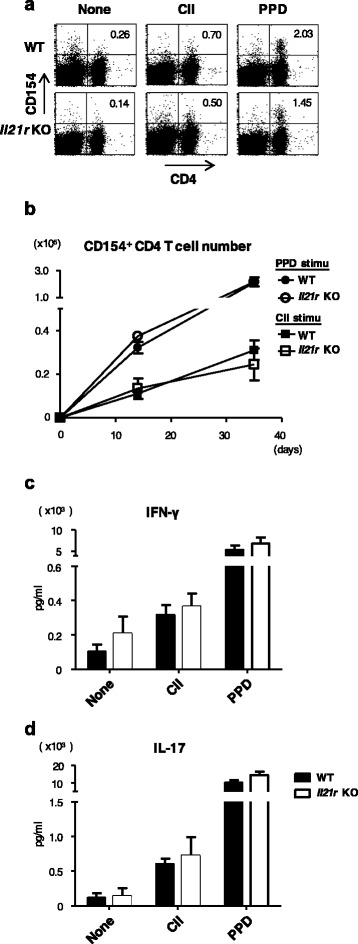
Antigen-specific CD4 T-cell responses were comparable between WT and *Il21r* KO mice with CIA. **a** Representative dot plot analysis of intracellular CD154 expression in WT (*upper*) or *Il21r* KO (*lower*) CD4 T cells 35 days after CIA induction. CD154 within CD4 T cells was stained after ex-vivo stimulation with nothing (*left*), CII (*middle*), or PPD (*right*). Numbers (*upper right quadrant*) indicate the percentage of CD154^+^ cells in CD4 T cells. **b** Absolute numbers of CD154^+^ CD4 T cells in WT mice (*filled symbols*) or *Il21r* KO mice (*open symbols*) during the course of CIA. IFN-γ (**c**) and IL-17 (**d**) secretion of draining LNs in WT mice (*filled column*) or *Il21r* KO mice (*open column*) 35 days after CIA induction. Both cytokines of supernatants were measured by ELISA after ex-vivo stimulation with CII (*middle*) or PPD (*right*). Error bars represent mean ± SEM. Statistical significance was analyzed by two-way ANOVA with repeated measures (**b**–**d**). Data are representative of three independent experiments. *CII* type II collagen, *IFN* interferon, *IL* interleukin, Il21r *KO* IL-21 receptor knockout, *PPD* purified protein derivative, *WT* wild type

We next examined CII-specific T-cell responses in *Il21r* KO mice. In-vivo clonal expansion of antigen-specific T cells was evaluated by detecting the intracellular expression of CD154 in CD4 T cells obtained from the draining LNs after brief stimulation with the antigens [25]. As shown in Fig. 4a, there was a clear population of CD154-positive cells after stimulation with CII or PPD, and there was no difference in the number of antigen-specific CD4 T cells between WT mice and *Il21r* KO mice (Fig. 4b). Since we could not detect antigen-specific CD4 T cells that produced IFN-γ or IL-17 by flow cytometric analysis, possibly because their frequency was too low, we examined the levels of cytokines secreted in the culture supernatants after stimulation with the antigens. There was no difference in antigen-specific IL-17 and IFN-γ production between WT and KO T cells (Fig. 4c, d). Consistent with this, we detected comparable levels of T-cell proliferation after in-vitro restimulation with the antigens by measuring ^3^H-thymidine uptake (data not shown).

### Normal Tfh cell development in *Il21r* KO mice

**Fig. 5:**
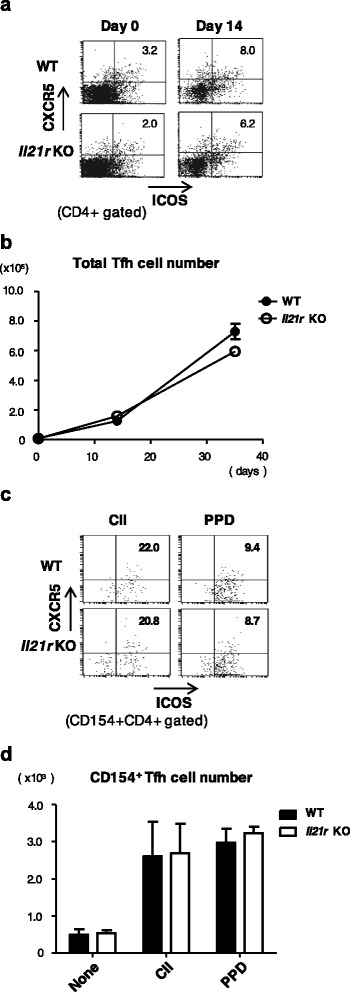
Development of Tfh cells was similar in WT and *Il21r* KO mice with CIA. **a** Representative dot plots of CXCR5 and ICOS expression on CD4 T cells 14 days after CIA induction. Numbers (*upper right quadrant*) indicate the percentage of CXCR5^+^ICOS^+^ cells in CD4 T cells. **b** Absolute numbers of Tfh cells (CXCR5^+^ICOS^+^CD4^+^ cells) in the draining LNs of WT mice (*filled circle*) or *Il21r* KO mice (*open circle*) during the course of CIA. **c** Representative dot plots of CXCR5 and ICOS expression in CD154^+^ CD4 T cells 14 days after CIA induction. Numbers (*upper right quadrant*) indicate the percentage of CXCR5^+^ICOS^+^ cells in CD154^+^ CD4 T cells. **d** Absolute numbers of CII-specific Tfh cells (CXCR5^+^ICOS^+^CD154^+^CD4^+^ cells) in the draining LNs of WT mice (*filled column*) or *Il21r* KO mice (*open column*) during the course of CIA. Error bars represent mean ± SEM. Statistical significance was analyzed by two-way ANOVA with repeated measures (**b**, **d**). Data are representative of three independent experiments. *CII* type II collagen, Il21r *KO* IL-21 receptor knockout, *PPD* purified protein derivative, *Tfh* follicular helper T, *WT* wild type

The development of Tfh cells in WT mice and *Il21r* KO mice was examined by detecting CD4 T cells expressing CXCR5 and ICOS. Although IL-21 was reported to be involved in the development of Tfh cells [4], there was no difference in the frequency or number of Tfh cells between WT mice and *Il21r* KO mice (Fig. 5a, b). We examined the development of antigen-specific Tfh cells by analyzing cell populations that expressed CD154 after in-vitro antigenic stimulation. There was no difference in the number of CII-specific or PPD-specific Tfh cells between WT mice and *Il21r* KO mice (Fig. 5c, d). We could not detect CII-specific or PPD-specific IL-21-producing T cells either by flow cytometric analysis or ELISA (data not shown). IFN-γ or IL-17 production by Tfh cells was not observed (data not shown).

### Importance of IL-21-R on B cells in the development of CIA

**Fig. 6:**
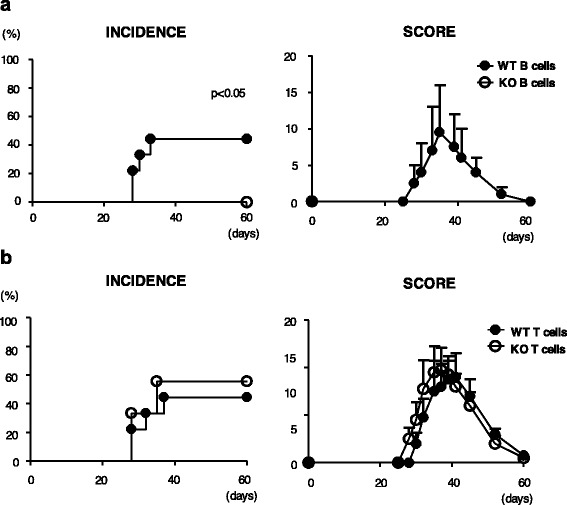
Development of CIA in *Rag2* KO mice reconstituted with WT and *Il21r* KO T cells and B cells. **a** *Rag2* KO mice were transferred either with WT CD4 T cells and WT B cells (*WT B cells*), or with WT CD4 T cells and *Il21r* KO B cells (*KO B cells*). **b** *Rag2* KO recipients were transferred either with WT CD4 T cells and WT B cells (*WT T cells*), or with *Il21r* KO CD4 T cells and WT B cells (*KO T cells*). The recipients were immunized with CII to induce CIA. Incidence (*left*) and severity score (*right*) of CIA are shown. Data are the sum of three independent experiments (*n* = 9). Clinical scores were calculated only in affected mice. Error bars represent mean ± SEM. Statistical significance was analyzed by the log-rank test (**a**, **b** *left*). *KO* knockout, *WT* wild type

The results described suggest that the lack of IL-21 signaling affected pathogenic autoantibody production of B cells without impairing T-cell functions. To confirm whether the lack of IL-21 signaling in B cells is sufficient for preventing the development of CIA, we conducted adoptive transfer experiments in which B cells and T cells from WT mice or *Il21r* KO mice in different combinations were transferred into *Rag2* KO recipient mice. In the first set of experiments, *Rag2* KO mice were transferred with B cells from naïve WT mice or *Il21r* KO mice together with CD4 T cells from CII-immunized WT mice. The recipients were then immunized with CII to induce CIA. We found that the recipients to which WT B cells were transferred developed arthritis, whereas those receiving KO B cells did not (Fig. 6a). In the other set of experiments, donor CD4 T cells were transferred either from WT mice or *Il21r* KO mice, but CIA developed in both recipients as long as they had been transferred with WT B cells (Fig. 6b). These data indicate that IL-21 signaling in B cells, but not in T cells, is indispensable for the development of CIA.

## Discussion

In the present study, we demonstrated that *Il21r* KO mice were highly resistant to the development of CIA, due to the lack of IL-21 signaling in B cells which is essential for the production of pathogenic anti-CII autoantibody. Several studies have examined the roles of IL-21 in animal models of RA. Young et al. [23] examined the effect of blocking IL-21 in the effector phase of CIA by administering IL-21R-Fc after the onset of arthritis. They observed a reduction of disease severity, but the level of CII-specific Ab was not reduced. IL-21 blockade enhanced the production of IFN-γ, IL-2, and GM-CSF, but reduced IL-17 and IL-6 production by splenocytes [23]. However, as the treatment started at the effector stage of the disease, these changes could be a consequence, but not necessarily the cause, of the reduced inflammation. In this regard, Block and Huang [26] made a detailed observation of the roles of IL-21 signaling in immune responses in the K/BxN mouse model of spontaneous arthritis. Similar to our results, they demonstrated that IL-21R on B cells was sufficient for the development of arthritis. Their observation was made in cell transfer experiments, however, and IL-21 might play an additional role in the spontaneous development of arthritis in K/BxN mice. In normal conditions, homeostatic expansion of KRN TCR Tg T cells is a critical step for disease development, and IL-21 was shown to be involved in this process [27]. In contrast, in the case of CIA, CII-specific T cells are assumed to expand after immunization with CII using IL-2 as the growth factor, which is produced upon Ag recognition by TCR.

The roles of IL-21 signaling in different immune cell compartments have also been addressed in other models of autoimmune diseases. For instance, McPhee et al. [28] showed that IL-21 signaling in B cells, but not in T cells, was required for the development of systemic lupus erythematosus-like disease in BXSB mice using mixed bone-marrow chimera mice. The essential role of IL-21 signaling in B cells is thus not limited to the pathogenesis of autoimmune arthritis.

Our data indicate that IL-21 signaling in T cells is dispensable not only for the development of CIA but also for the development of Th17 and Tfh cells. However, earlier studies showed the role of IL-21 signaling in the development of these CD4 T-cell subsets [3, 29]. Although we cannot explain the reason for this discrepancy, similar to our results, several studies have indicated that IL-21 signaling was dispensable for the development of Th17 cells [30, 31]. In addition, Th17 cells increased in *Il21r* KO K/BxN mice [27], and it was later demonstrated that Th17 cells were not even required development of the disease [26]. Although IL-21-induced STAT3 activation is involved in the development of Th17 cells via the induction of RORγt, IL-6 alone might induce sufficient levels of STAT3 activation. In fact, IL-6 production is enhanced in mice with CIA [32]. Similar to Th17 cells, Tfh cells were shown to develop normally in *Il21* KO mice and *Il21r* KO mice [5, 33]. Although the molecular mechanisms of Tfh development are not fully understand, the engagement of TCR with high-affinity ligand and ICOS signaling were shown to be involved in the development of Tfh cells, in addition to STAT3-activating signals such as IL-6 and IL-21 [34, 35].

IL-21, which is produced by Tfh cells, plays a critical role in the differentiation and proliferation of GC B cells [6] through the regulation of Bcl-6 expression in B cells [5]. Consistent with this, *Il21r* KO mice showed the impaired production of antigen-specific IgG1 [7]. Therefore, it could be expected that IL-21 signaling in B cells is also involved in CII-specific IgG production required for the development of CIA. On the contrary, we did not detect a difference in the levels of CII-specific IgM production between WT mice and *Il21r* KO mice. This might be because IL-21 signaling is not involved in the activation of CII-specific naïve B cells first primed at the border of T-cell and B-cell areas. Alternatively, such IgM, if not all, is produced via an extrafollicular pathway such as in MZ B cells. These results indicate that the development of CIA depends on GC-derived autoantibodies, although it is currently unclear whether isotype class switching or affinity maturation, or both, is important for disease development.

IL-21 has been implicated in the pathogenesis of RA and therefore is an emerging therapeutic target [24]. In fact, clinical trials targeting IL-21 for the treatment of RA are ongoing [36]. Our data suggest that targeting IL-21 might reduce autoimmune pathology induced by autoantibodies, while preserving T-cell responses for host defense against pathogens. It is also noteworthy that affinity maturation of ACPA precedes the onset of RA [37], raising a possibility that blocking IL-21 signaling at this phase might prevent the onset of the disease. Although it is currently difficult to precisely identify those individuals at risk of developing RA, this is an emerging topic [38]. Further investigation of the functions of IL-21 in immune systems, especially in humans, is required for the development of an optimal IL-21-targeting therapy for RA.

## Conclusions

We demonstrated in this study that IL-21 signaling in B cells, but not in T cells, is involved in the development of CIA, via the induction of pathogenic autoantibodies against CII. IL-21 thus affects both the initiation and the progression of autoimmune arthritis.

## Abbreviations

Ab: antibody
ACPA: anti-citrullinated protein antibody
CFA: Freund’s complete adjuvant
CIA: collagen-induced arthritis
CII: chicken type II collagen
ELISA: enzyme-linked immunosorbent assay
GC: Germinal Center
IFN-γ: interferon gamma
IL: interleukin
IL-21R: interleukin-21 receptor
*Il21r* KO: interleukin-21 receptor knockout
LN: lymph node
NKT: natural killer T
PPD: purified protein derivative
RA: rheumatoid arthritis
RF: rheumatoid factor
Tfh: follicular helper T
Th17: T-helper cell type 17
WT: wild type
